# Multiple novel gene-by-environment interactions modify the effect of *FTO* variants on body mass index

**DOI:** 10.1038/ncomms12724

**Published:** 2016-09-06

**Authors:** Alexander I. Young, Fabian Wauthier, Peter Donnelly

**Affiliations:** 1 Wellcome Trust Centre for Human Genetics, University of Oxford, Oxford OX3 7BN, Oxfordshire, UK; 2 Department of Statistics, University of Oxford, 24–29 St Giles', Oxford OX1 3LB, UK

## Abstract

Genetic studies have shown that obesity risk is heritable and that, of the many common variants now associated with body mass index, those in an intron of the fat mass and obesity-associated (*FTO*) gene have the largest effect. The size of the UK Biobank, and its joint measurement of genetic, anthropometric and lifestyle variables, offers an unprecedented opportunity to assess gene-by-environment interactions in a way that accounts for the dependence between different factors. We jointly examine the evidence for interactions between *FTO* (rs1421085) and various lifestyle and environmental factors. We report interactions between the *FTO* variant and each of: frequency of alcohol consumption (*P*=3.0 × 10^−4^); deviations from mean sleep duration (*P*=8.0 × 10^−4^); overall diet (*P*=5.0 × 10^−6^), including added salt (*P*=1.2 × 10^−3^); and physical activity (*P*=3.1 × 10^−4^).

## Abstract (web summary)

Common variants in the fat mass and obesity associated (*FTO*) gene are linked to body mass index (BMI). Using the latest UK Biobank data, this study shows novel gene x environment interactions between *FTO* and lifestyle factors, including frequency of alcohol consumption, sleep, and added dietary salt.

## Graphical abstract

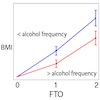

The obesity epidemic is causing a growing burden on public health^1^. Body mass index (BMI), defined as weight divided by height squared, is the most commonly used measure of adiposity, with individuals exceeding a certain BMI threshold classed as obese^2^. BMI, such as obesity, is positively correlated with metabolic abnormalities, many common diseases and all-cause mortality^3^. While the principal causes of the obesity epidemic may be environmental at a societal scale, studies have shown that genetic differences underlie much of the variation in BMI between individuals^4^. Understanding the genetic risk factors, as well as the way in which they interact with environmental risk factors, can thereby give new insights into the biology underlying obesity.

The common genetic variants with the largest effect on BMI variation between individuals are located in an intron of the fat mass and obesity-associated (*FTO*) gene^5^. In Europeans, each additional copy of the risk allele at single-nucleotide polymorphism (SNP) rs1558902, one of the cluster of associated SNPs, increases average BMI by between 0.35 and 0.43 kg m^−2^, explaining ∼0.34% of the variation in BMI^5^,^6^. The fact that the variants are located in an intron of the *FTO* gene does not establish that they act through that gene. Nevertheless, for convenience, we will follow other authors and refer to the locus and to the associated variants as ‘*FTO*', with specific SNPs referenced when relevant. While progress has been made in understanding the causal mechanisms through which the *FTO* risk alleles increase BMI, implicating regulation of expression of other genes^7^,^8^,^9^, the mechanisms and their interactions with lifestyle and environmental factors are not fully characterized.

There have been many small studies examining interactions between the *FTO* locus and various environmental and lifestyle variables. Results have often been inconsistent, especially when comparing studies across different cultures or ethnicities. One possible cause of the inconsistencies may be the difficulty of measuring environmental variables consistently across studies, but low power to detect interactions may also be a contributing factor. Nevertheless, large meta-analyses have found a reduction of the effect of *FTO* on BMI of ∼30% in physically active people^10^,^11^,^12^. *FTO* has also been linked to interactions with diet, especially fried and fatty foods^13^,^14^,^15^,^16^, but did not appear to interact with macronutrient intake or dietary energy in a meta-analysis^17^.

Meta-analyses typically involve some level of data aggregation within studies before combining across studies. Meta-analyses have usually proceeded by dichotomizing continuous or ordinal variables so as to reduce between-study heterogeneity. This leads to a loss of power compared with a similarly sized study using the original measurements^11^,^18^. Dichotomizing variables can also reduce the specificity and interpretability of results, which can reduce their utility for public health. The heterogeneity between different studied cohorts, in both measurement of the environment and genetic and cultural heterogeneity, can reduce power compared with a similarly sized single-cohort study^11^.

A major challenge in studying environmental risk factors is that many of these are highly correlated with each other. It is then unclear whether an observed interaction between *FTO* and an environmental variable might be driven by its correlation with other environmental variables. When they are simultaneously measured on the same individuals, fitting multiple interaction effects jointly can help determine whether the environmental variables interact with *FTO* independently of their correlations with each other. Power considerations, and/or lack of the appropriate data, have precluded the fitting of multiple interaction effects simultaneously in smaller studies, and large meta-analyses have typically analysed only one interaction effect at a time^10^,^11^,^17^. It is therefore unclear whether many of the interactions reported in the literature are truly independent of each other and of other variables.

The UK Biobank is a large prospective study of 500,000 individuals aged between 40 and 69 years at recruitment between 2006 and 2010. Extensive measurements and questionnaire responses, including rich lifestyle and environmental information, were gathered from individuals at baseline, and biological samples taken to allow additional assays^19^,^20^,^21^. The recent interim genetic data release includes genotype data on ∼152,000 of these individuals^22^,^23^. The UK Biobank therefore offers a unique opportunity to examine interactions between *FTO* and various lifestyle, and environmental variables simultaneously in a large and relatively homogeneous sample.

By joint modelling, we investigated interactions between *FTO* (specifically SNP rs1421085) and physical activity, frequency of alcohol consumption, dietary variation, sleep duration, smoking, TV watching, and socioeconomic status. We focussed on rs1421085 following a recent study suggesting this is the causal variant^7^. We note that rs1421085 is highly correlated with the main previously studied SNPs, in particular rs9939609 (*r*^2^=0.89). This facilitates comparison with previous interaction studies^10^, and we note that the results of our analyses are little changed if rs9939609 is used for the genetic effect.

We find evidence for novel interactions between *FTO* and frequency of alcohol consumption and deviations from mean sleep duration, with the effect of *FTO* diminishing with the frequency of alcohol consumption and increasing with deviations from average sleep duration. We estimate that dietary variation has the strongest interaction with *FTO* and make a novel observation that the effect of *FTO* on BMI is enhanced in those who add salt to food more frequently. Our joint modelling increases confidence that the interaction between *FTO* and physical activity is not due to confounding with other lifestyle variables in our model. These findings increase our understanding of how lifestyle modifies the effect of *FTO* on BMI, which may be indicative of more general mechanisms of relevance to the management of obesity genetic risk.

## Results

### UK Biobank samples

We first constructed three distinct subsets of individuals from the full UK Biobank sample with complete information for the requisite lifestyle and anthropometric variables (Methods). The first subset is a set of 231,906 individuals with self-declared British ancestry who were born in the United Kingdom or Ireland on whom genetic data were not available in the interim data release. The second subset is a set of 89,552 ‘British' individuals on whom genotype information was available and who passed sample QC and were identified in the UK Biobank QC analysis as being of self-declared British ancestry and for whom genome-wide genetic analyses confirmed northern-European ancestry. We pruned this subset of any individuals with third degree or closer relatives genotyped in the UK Biobank. The third subset is the remaining 29,580 individuals passing UK Biobank sample QC for whom genetic data is available, which we label the ‘Diverse Sample'. The self-declared ethnicities of this subset are reported in Supplementary Table 1. While 71% are of British and Irish ethnicity, there is considerable genetic diversity present (Supplementary Fig. 1), with ∼17% of the sample from a non-white background and 21% born outside the United Kingdom and Ireland.

We used the first of these subsets, which we refer to as the ‘non-genotyped sample' to learn about the relationship between lifestyle factors and BMI.

The reason for defining two separate groups among individuals with genotype data was to allow us to deal with the differing types of population structure present in a computationally efficient manner. Population structure affects genetic association testing when a trait differs in a systematic way between populations that differ genetically^24^. A similar effect can be caused by family relatedness between members of a sample^24^. Linear mixed models have been proven to be effective at controlling for both population structure and family relatedness^25^, although they are computationally expensive. Our split of the samples enabled us to only use a computationally expensive mixed model analysis in the smaller ‘Diverse' Sample to control for the complex structure present (Supplementary Note 1). The British Sample, in contrast, has weaker population structure and has been pruned of relative pairs, allowing us to employ principal component analysis (Methods), which has been proven effective at controlling for structure in UK samples without close relatedness^25^,^26^ and is computationally more efficient. The sample split also gives us some power to observe heterogeneity of effects between the British Sample and a more genetically and culturally Diverse Sample.

The baseline characteristics of the samples are recorded in Table 1. Height was measured at assessment centres by a Seca 240 cm height measure, and weight was measured using a Tanita BC418MA body composition analyser^27^.

### Construction of activity and diet scores

The UK Biobank contains many highly correlated lifestyle variables that affect BMI (Methods). To reduce dimensionality, we constructed a single summary variable, which we refer to as a ‘score', for physical activity and diet. We briefly outline the score construction in the following—see Methods for details.

The scores weight the different variables in a category (for example, physical activity) by the strength and direction of their association with BMI, while accounting for their correlations with other predictors of BMI. In brief, in the non-genotyped sample, we regressed log-BMI on all the variables in the categories together with other variables associated with BMI. We then used cross-validation to remove variables without any apparent predictive ability^28^, and refitted the model on the remaining variables. The fitted coefficients from this model for the variables in a particular category were then applied to those variables for each individual with genotype data to calculate the category score for that individual. The category score can be thought of as the best single predictor of BMI based on the variables in that category. Note that to avoid possible over-fitting, we estimated the coefficients used to calculate the score in a distinct set of individuals from those in which we actually calculated scores. The variables used to construct each score are listed in Table 2 under the ‘BMI' model.

### Modelling

We fitted models separately in the British and Diverse Samples and tested for heterogeneity between the samples using the standard Q statistic for heterogeneity in meta-analysis^29^. We combined estimates in a fixed effects meta-analysis using the *R*^30^ package ‘meta'^31^ if the *P* value for the heterogeneity test was above 0.05. We performed 25 interaction tests in total and considered an interaction significant if its *P* value was less than the Bonferroni-corrected significance threshold of 0.05/25=0.002. The same numbers of tests for main effects were performed, so we used the same significance threshold for these. We report the uncorrected *P* values.

Unless otherwise stated, we modelled BMI on the log-scale (Methods), and we report estimated effects transformed back onto the original scale, where we express them as a percentage change in BMI per s.d. of the relevant predictor. We give the 95% confidence intervals in square brackets.

**Figure 1:**
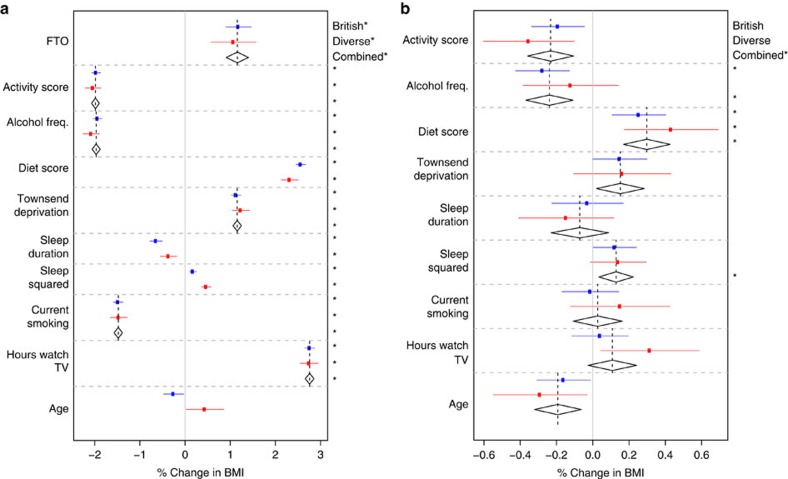
Main effects and interactions with FTO. The estimated (**a**) main effects on BMI (% change in BMI per risk allele for FTO, per decade for age and per s.d. for other variables) and (**b**) interaction effects with FTO on BMI (% change in BMI per FTO risk allele per decade for age and % change in BMI per FTO risk allele per s.d. for other variables). All main and interaction effects were fitted jointly in the ‘Scores' Model (Table 2) in both the British (*n*∼90,000) and Diverse (*n*∼30,000) Samples. The estimated effects are shown along with their 95% confidence intervals in both the British (blue) and Diverse (red) Samples along with the combined estimate from a fixed effects meta-analysis when no significant heterogeneity between samples was observed (diamonds). ‘Sleep Squared' refers to squared deviations from mean sleep duration. A star on the right indicates a *P* value below the Bonferroni-corrected significance threshold of 0.05/25=0.002.

**Figure 2:**
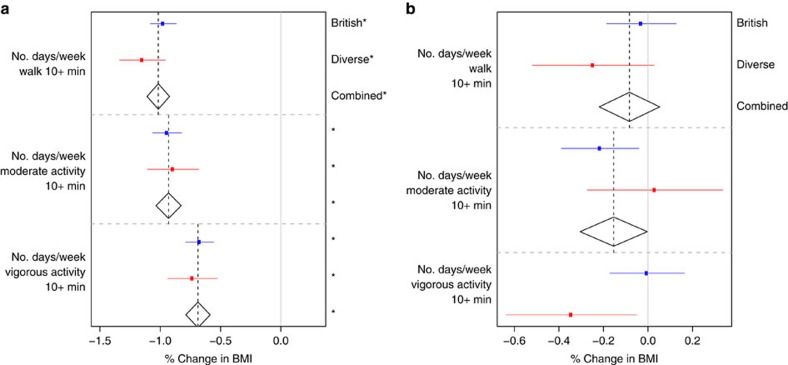
Main effects and interactions with FTO of activity variables. For the components of the activity score, the estimated (**a**) main effects on BMI (% change in BMI per s.d.) and (**b**) interaction effects with FTO on BMI (% change in BMI per FTO risk allele per s.d.). All main and interaction effects were fitted jointly in the ‘Activity' Model (Table 2) in both the British (*n*∼90,000) and Diverse (*n*∼30,000) Samples. The estimated effects are shown along with their 95% confidence intervals in both the British (blue) and Diverse (red) Samples along with the combined estimate from a fixed effects meta-analysis when no significant heterogeneity between samples was observed (diamonds). A star on the right indicates a *P* value below the Bonferroni-corrected significance threshold of 0.05/25=0.002.

**Figure 3:**
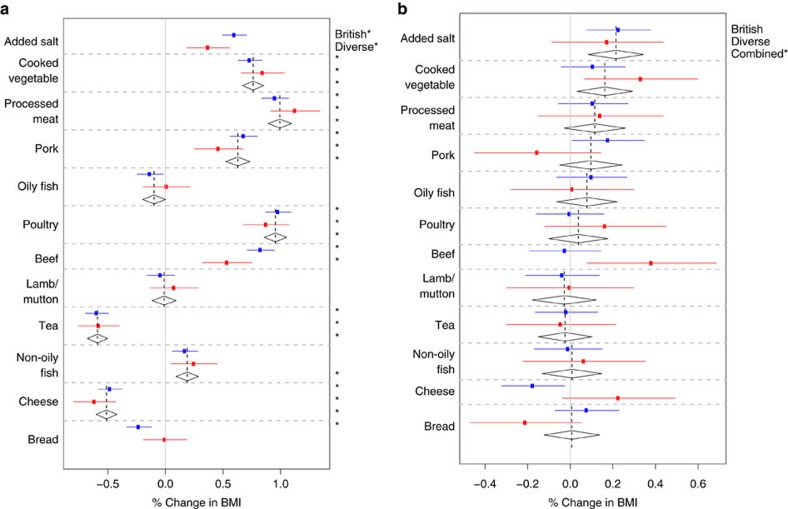
Main effects and interactions with FTO of dietary variables. For the components of the diet score, the estimated (**a**) main effects on BMI (% change in BMI per s.d.) and (**b**) interaction effects with FTO on BMI (% change in BMI per FTO risk allele per s.d.). All main and interaction effects were fitted jointly in the ‘Diet' Model (Table 2) in both the British (*n*∼90,000) and Diverse (*n*∼30,000) Samples. The estimated effects are shown along with their 95% confidence intervals in both the British (blue) and Diverse (red) Samples along with the combined estimate from a fixed effects meta-analysis when no significant heterogeneity between samples was observed (diamonds). A star on the right indicates a *P* value below the Bonferroni-corrected significance threshold of 0.05/25=0.002.

Our primary analyses involved fitting the ‘Scores Model' (Table 2) that jointly models all main effects of BMI-associated variables, including the activity and diet scores, and their interactions with *FTO*. We report effects from fitting this model (Fig. 1), and additionally we report main and interaction effects for the components of the activity (Fig. 2) and diet (Fig. 3) scores from the ‘Activity' and ‘Diet' Models (Table 2). Where there is no strong evidence for heterogeneity of effects between samples, we report only the combined estimate. Table 3 gives a statistical summary of the estimated effects.

### FTO

In the Scores Model (Table 2), we estimate that each additional copy of the rs1421085 risk allele is associated with a BMI increase of 1.17% in the British Sample ([0.90%, 1.44%], *P*=1.0 × 10^−17^) and 1.07% in the Diverse Sample ([0.58%, 1.57%], *P*=2.2 × 10^−5^). There is no evidence for heterogeneity (*P*=0.73), with a combined estimate of 1.15% ([0.91%, 1.38%], *P*=1.22 × 10^−21^; Fig. 1a). To compare with earlier studies, we also fitted the Scores Model (Table 2) on untransformed BMI in the British Sample, giving an estimated additive effect of 0.34 kg m^−2^, with a 95% confidence interval of [0.26 kg m^−2^, 0.41 kg m^−2^]. This is in agreement with a previous meta-analysis (total *N* ∼250,000) estimate of 0.39 kg m^−2^ for rs1558902, another *FTO* SNP in strong linkage disequilibrium with rs1421085 (refs 5, 6).

### Physical activity

The activity score is associated with a reduction in BMI of 1.98% ([−2.07%, −1.90%], *P*<10^−30^) per s.d., and there is strong evidence that all the individual activity variables are also associated with reduced BMI in each sample (Fig. 2a). We found that *FTO* interacts with the activity score (−0.23% [−0.36%, −0.11%], *P*=3.1 × 10^−4^), with *FTO* having a weaker effect in more physically active individuals.

Physical activity is the variable with the strongest prior evidence for an interaction with *FTO*^10^,^11^. There is strong evidence for an interaction between FTO and physical activity from meta-analyses of North American cohorts and combined European and North American Cohorts. Some but not all individual studies in European cohorts have found statistically significant interactions between physical activity and *FTO* on BMI^32^,^33^,^34^,^35^,^36^,^37^. Large meta-analyses have not found statistically significant interactions between *FTO* and physical activity when restricted to European cohorts^10^,^11^. To aid comparison with a previous meta-analysis^10^, we fitted the ‘Scores' Model (Table 2) on untransformed BMI with the activity score dichotomized at its 20th percentile. Our estimate for the interaction between physical activity and *FTO* on BMI in this model is −0.19 kg m^−2^ difference in per copy *FTO* effect ([−0.29 kg m^−2^, −0.08 kg m^−2^], *P*=0.001), which is larger (*P*=0.075 for difference) than a meta-analysis estimate of European cohorts (*n*=164,307: −0.06 kg m^−2^, [−0.16, 0.03] kg m^−2^, *P*=0.18)^10^. We note that the estimate is very close to the estimate from the European Prospective Investigation of Cancer (EPIC) Norfolk cohort reported in the meta analysis (−0.18 kg m^−2^ [−0.34 kg m^−2^, −0.02 kg m^−2^])^10^, which is a cohort of similar genetic, cultural and age composition^32^ to the UK Biobank. Our estimate is thus intermediate between a meta-analysis of North American cohorts estimate (*n*=47,938: −0.49 kg m^−2^ [−0.65,−0.33] kg m^−2^) and the meta-analysis of European cohorts estimate^10^ and is consistent with the EPIC Norfolk estimate. Our results support the picture in the literature of a larger interaction effect in North American cohorts compared to European cohorts.

### Alcohol consumption

Frequency of alcohol consumption is associated with a decrease in BMI of 1.97% per s.d. (95% confidence interval [−2.06%, −1.88%], *P*<10^−30^; Fig. 1a). This is in agreement with previous studies that have observed the number of days per week that an individual drinks alcohol is inversely associated with BMI, whereas total alcohol intake is positively associated^38^,^39^.

While *FTO* has been shown to affect alcohol consumption patterns^40^, alcohol consumption patterns have not been previously found to modify the effect of *FTO* on BMI. We found that the effect of *FTO* on BMI is diminished with more frequent consumption of alcohol (−0.24% [−0.37%, −0.11%], *P*=3.0 × 10^−4^).

### Diet score

The diet score was associated with an increase in BMI of 2.56% per s.d. in the British Sample ([2.45%, 2.67%], *P*<10^−30^) and 2.32% ([2.13%, 2.50%], *P*<10^−30^) in the Diverse Sample, with some evidence for heterogeneity (*P*=0.024). To better understand which nutritional properties of diet were driving the association between diet score and BMI, we took advantage of a small subsample (∼12,500 in the British Sample; ∼4,500 in the Diverse Sample) of people who had a 24-h diet recall questionnaire administered, from which UK Biobank estimated nutrient quantities (Methods)^41^. Protein, food weight and saturated fat all had strong positive associations with both BMI and the diet score.(Supplementary Fig. 2).

We found that the effect of *FTO* on BMI is enhanced in individuals with a higher diet score (0.30% ([0.17%, 0.43%], *P*=5.0 × 10^−6^), the strongest estimated *FTO* interaction effect in the joint model.

### Dietary components

We investigated the relationship between specific components of the diet score and BMI (Fig. 3a). We found that how frequently one adds salt to food (added salt) was associated with increased BMI in the British Sample (0.60% [0.49%, 0.71%], *P*<10^−30^) and the Diverse Sample (0.37% [0.19%, 0.56%], *P*=7.5 × 10^−5^), with some evidence for heterogeneity (*P*=0.034). Added salt was associated with food energy estimated from 24-h diet recall (*P*=1.2 × 10^−3^; Supplementary Fig. 3).

Cooked vegetable intake is consistently associated with increased BMI in both the British and Diverse Samples (combined estimate: 0.76% [0.67%, 0.85%], *P*<10^−30^). In the detailed nutrient study, cooked vegetable intake is associated with increased protein, carbohydrate and food weight (Supplementary Fig. 3), which are all associated with increased BMI (Supplementary Fig. 2), possibly explaining the positive association between cooked vegetable intake and BMI.

We investigated whether there was evidence for an interaction between *FTO* and any of the 12 variables comprising the diet score (Fig. 3b). The strongest evidence for any particular dietary variable interacting with *FTO* is for added salt (0.21% [0.08%, 0.34%], *P*=1.2 × 10^−3^); the effect of FTO is increased for individuals who add salt to food more frequently.

We also tested for interactions between *FTO* and the estimated nutrient quantities for the subset of the British Sample for whom a 24-h dietary recall questionnaire had been administered (*n*∼12,500, so substantially less powered than our main analyses), and we did not find any statistically significant evidence for interactions (data not shown).

### Sleep

A non-linear U-shaped relationship between sleep duration and BMI has been observed^42^. We fitted both sleep duration and the squared deviation from mean sleep duration as effects on BMI, finding that the linear term is associated with reduced BMI (British: −0.48% [−0.58%, −0.38%], *P*=7.7 × 10^−20^; Diverse: −0.36% [−0.55%, −0.18%], *P*=1.1 × 10^−4^; Fig. 1a), while the squared deviation is associated with increased BMI (British: 0.42% [0.36%, 0.49%], *P*<10^−30^; Diverse: 0.47% [0.36%, 0.58%], *P*=4.7 × 10^−18^). This is in agreement with previous studies showing that more sleep is associated with lower BMI in a small range around the average, while large deviations from the average amount of sleep are associated with increased BMI.

There was no evidence that linear variation in sleep duration modifies the effect of *FTO* on BMI (−0.02% [−0.17%, 0.13%], *P*=0.43), whereas there was evidence that increases in the squared deviation from mean sleep enhance the effect of *FTO* on BMI (0.13% [0.06%, 0.21%], *P*=8.0 × 10^−4^).

### Townsend deprivation index

Lower socioeconomic status has been shown to be associated with higher BMI in developed countries^43^. The Townsend deprivation index is a combined measure of indicators of socioeconomic deprivation in a geographic region^44^, and in our data it is associated with increased BMI (1.15% [1.06%, 1.25%], *P*<10^−30^). The estimated interaction between FTO and Townsend deprivation index was not significant after Bonferroni correction (*P*=0.035).

### Age

We found evidence that age is associated with reduced BMI in the joint model in the British Sample (−0.25% per decade [−0.48%, −0.03%], *P*=0.026; Fig. 1a), although its univariate correlation with BMI is positive. In the Diverse Sample, age is associated with increased BMI in the joint model (0.44% per decade [0.02%, 0.86%], *P*=0.040 in the Diverse Sample). The age range in the genotyped sample is between 39 and 70, so the effect of age estimated here reflects the difference between middle and older age, and is not informative for ages outside this range. While we saw evidence for an interaction between FTO and age this was not significant after Bonferroni correction (*P*=0.006).

### TV watching

TV watching has been shown to strongly correlate with BMI^45^, and does so in our data (2.76% [2.66%, 2.85%], *P*<10^−30^). In contrast to a previous study^45^, we do not find strong evidence for an interaction between TV watching and *FTO* (0.11% [−0.03%, 0.24%], *P*=0.15). However, we note that fitting a non-joint model with only the interaction between TV and *FTO* in the British Sample results in a much more statistically significant interaction estimate (0.21% [0.07%, 0.35%], *P*=0.003), demonstrating that joint interaction modelling can prevent overestimation of interaction effects in the presence of multiple correlated lifestyle factors.

### Current smoking

While there is evidence that being a current regular smoker is associated with having a lower BMI than otherwise (−1.48% [−1.57%, −1.39%], *P*<10^−30^), there is no evidence that the effect of *FTO* is different between current regular smokers and others (0.03% [−0.11%, 0.16%], *P*=0.69).

### Robustness of interaction effects

We undertook various sensitivity analyses to examine how robust the interaction effect estimates were to potential sources of bias and confounding (Methods).

First, we tested whether our results were sensitive to exclusion of individuals with depression or diabetes and found they were not.

We analysed data on only one time point, so the effects we estimated between BMI and lifestyle variables could have been partially caused by behavioural modifications in response to changes in BMI—instances of ‘reverse causation'. Reverse causation would only generate a statistical interaction between a lifestyle factor and *FTO* on BMI if changes in the lifestyle factor in response to BMI depend on *FTO* genotype. We had limited power to address this issue, but there was information in participants' responses to questions about changes in dietary and alcohol consumption in the last 5 and 10 years, respectively. We found no evidence that the interaction effects were driven by recent changes in diet and alcohol consumption (Methods). However, by analysing the effect of FTO on lifestyle variables before and after correcting for BMI (Methods and Supplementary Table 2), we did find evidence that alcohol consumption may be decreased in response to increased BMI (Methods).

Given that all of the environmental variables we tested for interactions with *FTO* are related to overall health, we added self-rated overall health and its interaction with FTO to the models to test whether any of these interaction effects were being driven by their correlation with overall health (Methods). The interaction effects estimates were barely changed by this, arguing against major confounding with overall health.

## Discussion

**Figure 4:**
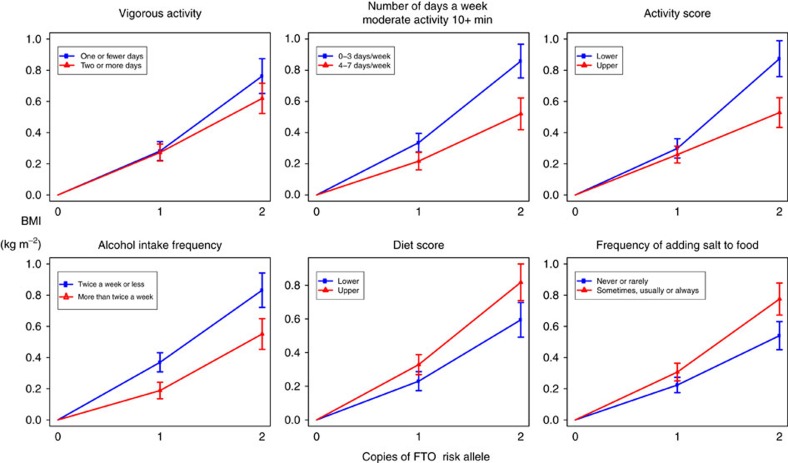
Modification of FTO effect by lifestyle. The effect of the FTO risk allele for different levels of different lifestyle variables in the British subsample. We split each lifestyle variable into two roughly equally sized categories. For each category, we plot the mean BMI and its 95% confidence interval for one and two copies of the FTO risk allele relative to zero copies. If there is no interaction between FTO and the environmental variable, the effect of adding another copy of FTO should be the same whatever the value of the environmental variables, and the lines for different categories should have the same gradient. If there is an interaction, they should diverge.

We have jointly analysed interactions on BMI between a variant in the first intron of the *FTO* gene (rs1421085) and several environmental and lifestyle factors. We undertook these joint analyses separately in two subsets of the UK Biobank data, a large (n∼89,500) ‘British' Sample, and a somewhat smaller (*n*∼29,500) ‘Diverse' Sample. We found evidence that *FTO* interacts with physical activity, frequency of alcohol consumption, dietary variation and squared deviations from mean sleep duration (Figs 1b, 2b, 3b and 4; Table 3). We did not find statistically significant evidence for interactions with current smoking status, Townsend deprivation index, age and TV watching. As our data relates mainly to individuals of European ancestry living in the United Kingdom, the results may not extend to other populations in different environments, or to children and adolescents.

As previous authors have argued^46^, there are major advantages in being able to assess main and interaction effects in the context of joint models that simultaneously include many potential predictors and covariates. While preferable, this approach has often not been possible in many earlier studies, either because a broad set of lifestyle factors have not been measured on study participants, or in the context of meta-analyses, because individual-level data are not available^11^,^18^. In our data, we found that testing only one interaction at a time would have led to a large overestimation of the interaction between *FTO* and TV watching, potentially leading to a statistically significant result that is not present in the joint model.

Very large resources such as UK Biobank that simultaneously measure genetic, lifestyle and phenotypic information thus offer substantial promise to further our understanding of gene × environment interactions. In our study, the joint interaction modelling gives us confidence that the interactions we find with physical activity, frequency of alcohol consumption, dietary variation and squared deviations from mean sleep duration are not due to confounding with each other and with variables correlated with age, socioeconomic status, current smoking and TV watching. This is an advantage over previous meta-analyses that have tested only one interaction at a time.

All of the diet and lifestyle variables we analysed in the UK Biobank data (but not BMI) were self-reported. While we cannot exclude that self-reporting affected our results, we think this unlikely: while self-reported data may be noisy and biased, it can only lead to spurious interactions with *FTO* on BMI if self-reporting as a function of BMI depends on *FTO* genotype, a phenomenon that seems *a priori* unlikely. The individual components of the activity and diet scores could be viewed as noisy observations of underlying latent ‘activity' and ‘diet' factors affecting BMI that may interact with *FTO*. Our construction and use of a single summary ‘score' from variables in these categories can be seen as a way of estimating these latent factors, helping to overcome the lack of precision in individual component measurements.

Evidence for an interaction between *FTO* and physical activity has been reported in several US-based studies, with the interaction estimated to be smaller in European cohorts^10^. We found evidence for a stronger interaction between *FTO* and physical activity than suggested by meta-analysis in European cohorts alone, but similar in magnitude to the interaction in another large British cohort (EPIC Norfolk)^10^,^12^. We estimated an interaction effect of −0.23% per activity score s.d. per copy of *FTO* risk allele. For BMI of 25 kg m^−2^, this represents a change of 0.41 kg m^−2^ per copy of *FTO* for −2 s.d. activity score versus 0.17 kg m^−2^ for +2 s.d. activity score, more than a halving of the original *FTO* effect. Care must be taken in interpreting this and earlier published results due to the fact that physically active people with higher BMIs may be more muscular than physically inactive people with equivalent BMIs.

We saw strong evidence of an interaction on BMI between *FTO* and diet score, which reflects variation in intake of 12 different variables. The combined estimate was 0.30% per s.d. per *FTO* risk allele; for BMI=25 kg m^−2^, this represents a change of 0.44 kg m^−2^ per copy of *FTO* for +2 s.d. diet score versus 0.17 kg m^−2^ for −2 s.d. diet score, more than doubling the *FTO* effect. The estimated effect was not stronger in those reporting dietary change in the past 5 years, reducing the chance it is due primarily to reverse causation. The interaction between FTO and diet score was the strongest interaction with FTO in our model.

We found evidence that the effect of *FTO* on BMI is enhanced in those who add salt to food more frequently: combined estimate of 0.21% per s.d. per *FTO* risk allele; for BMI of 25 kg m^−2^, this represents a change of 0.25 kg m^−2^ per copy of *FTO* for those who never or rarely add salt versus 0.43 kg m^−2^ for those who always add salt. More frequent addition of salt to food is associated with increased energy intake (Supplementary Fig. 3). It is plausible that adding salt to energy dense foods increases their palatability and therefore intake, and that this effect may be stronger in *FTO* risk allele carriers and those at the risk of obesity^47^.

We find strong evidence that greater frequency of alcohol consumption is associated with decreased BMI in both samples (Fig. 1a), which is in agreement with previous studies^38^,^39^, which have also shown a positive association between total alcohol consumption and BMI. Both these findings have been reported repeatedly, and so are likely real, even if they appear difficult to reconcile. Data on total alcohol consumption were only available for a small minority of our sample, preventing a joint analysis with the frequency of alcohol consumption. As far as we are aware, the only previous study reporting a gene–alcohol interaction for obesity found evidence that genetic risk for greater central abdominal fat, defined using a twin design, was reduced by greater alcohol consumption within the moderate range (*P*<0.05)^48^. Here we report evidence that the effect of *FTO* on BMI is reduced in more frequent consumers of alcohol. The combined estimate is −0.24% per s.d. per *FTO* risk allele; for BMI of 25 kg m^−2^, this represents a change of 0.33 kg m^−2^ per copy of *FTO* for those who drink two to three times a month versus 0.21 kg m^−2^ for those who drink daily or almost daily. There was almost no difference in the estimated interaction in those reporting no change in alcohol consumption over the past 10 years compared with those reporting a change, increasing confidence that the result is not primarily due to reverse causation. However, we did find evidence that *FTO* reduces alcohol consumption frequency, in agreement with previous studies^40^, possibly as a response to increased BMI. We therefore cannot rule out the possibility that the interaction we observe is due to a greater reduction of alcohol consumption frequency in response to higher BMI in *FTO* risk allele carriers compared with non-carriers. Nevertheless, our results highlight the need to further investigate the complex and statistically important relationship between alcohol consumption patterns, BMI and *FTO*.

The heritability of BMI has been observed to be higher in people who sleep <7 h a night compared with those that sleep >9 h a night (*P*<0.05)^49^, implying the genetic effects on BMI differ depending on sleep. We found evidence that squared deviations from mean sleep duration are associated with an enhanced effect of *FTO* on BMI (combined estimate: 0.13% per s.d. per FTO risk allele; for BMI of 25 kg m^−2^, this represents a change of 0.42 kg m^−2^ per copy of FTO for those who sleep 2 h more or less per night than the average versus 0.29 kg m^−2^ for the average, 7.16 h per night).

In common with other studies, it is possible that all the *FTO*–lifestyle interactions we report are driven by unobserved latent factors with which they are correlated. One possible confounder is overall health. We tried to control for this using a self-reported measure of overall health. The interaction effect estimates reduced only slightly in magnitude when self-reported overall health was included in the model, increasing confidence they are not simply reflecting an interaction with an underlying factor related to overall health. While controlling for factors such as overall health can increase confidence that an observed gene–lifestyle interaction is not due to a hypothesized confounding, there remains a need for randomization-based methods to demonstrate the causality of *FTO*–lifestyle interactions.

## Methods

### Selection of environmental variables

The variable names here are taken verbatim from the UK Biobank release, and information on them can be viewed in the UK Biobank Data showcase^50^.

Out of 17 continuous and ordinal dietary intake variables, we selected those without a large amount of missing data in the genotyped sample, where we count those who chose not to answer the question or did not know the answer as missing. This left 12 variables, 9 of which were ordinal (‘Oily fish intake', ‘Non-oily fish intake', ‘Processed meat intake', ‘Poultry intake', ‘Beef intake', ‘Lamb/mutton intake', ‘Pork intake', ‘Cheese intake' and ‘Salt Added to Food') and 3 of which were continuous (‘Cooked vegetable intake', ‘Bread intake' and ‘Tea intake').

All of the ordinal variables apart from ‘Salt Added to Food' were encoded as: 0, never; 1, less than once a week; 2, once a week; 3, 2–4 times a week; 4, 5–6 times a week; 5, once or more daily. ‘Salt Added to Food' uses the encoding: 1, never/rarely; 2, sometimes; 3, usually; 4, always. We found no strong evidence for anything beyond a linear association between log-BMI and the encoding of ‘Salt added to food'. We refer to ‘Salt added to food' as ‘added salt' for convenience.

For the remaining continuous intake variables, we excluded individuals who had values above the 99th percentile of the distribution in each sample to prevent being overly influenced by outliers.

We chose physical activity variables with near-complete observations: ‘Number of days/week walk 10 min or more', ‘Number of days/week of moderate physical activity 10+ minutes' and ‘Number of days/week of vigorous physical activity 10+ minutes'.

To prevent the loss of power, we selected the one alcohol intake variable with near-complete data, ‘alcohol intake frequency', which is encoded as: 0, never; 1, special occasions only; 2, one to three times a month; 3, once or twice a week; 4, three or more times a week; and 5, daily or almost daily. We compared the model fit of the regression of log-BMI on the original encoding of alcohol with the model fit when alcohol is encoded using the midpoint of implied monthly drinking sessions in each category. We found the model with the original encoding fitted better, so we retained the original encoding.

We used individuals' answers to the question ‘About how much sleep do you get in every 24 h? (please include naps)', and we excluded individuals in the bottom and top percentiles of the distribution to prevent being overly influenced by outliers. We enabled the fitting of the observed ‘U'-shaped relationship between BMI and sleep duration by also calculating the squared deviations from the mean sleep duration for each individual.

Townsend deprivation index was calculated immediately before participants joining UK Biobank based on the preceding national census output areas. Each participant was assigned a score corresponding to the output area in which their postcode is located.

Individuals' current smoking status was summarized by UK Biobank as ‘Never', 'Previous' and ‘Current'. For simplicity, we created a binary variable reflecting whether they answered ‘Current' or not.

We took individuals' answers to the question ‘In a typical day, how many hours do you spend watching TV? (put 0 if you do not spend any time doing it)', and we excluded those in the upper percentile of the distribution to avoid being overly influenced by outliers. There was an option to put ‘Less than an hour a day', which for definiteness we encoded as 0.5 h a day.

We used north and east co-ordinates (latitude and longitude) of place of birth in the United Kingdom as covariates to control for population stratification in the non-genotyped sample. In the genotyped sample, these were dropped in favour of genetic measures, namely, principal components or a mixed model (see below).

### Modelling

We log-transform BMI. This can be supported by the fact that BMI is restricted to be positive and fits a log-normal distribution better than a normal distribution (Supplementary Fig. 4). In addition, the effect of *FTO* on BMI can be modelled slightly better on the log scale as can be seen by fitting models for BMI and log-BMI in the British Sample with *FTO*, age, sex, age^2^, the confounding variables and the top 20 principal components as covariates: the variance explained is 6.8% for BMI, whereas for log-BMI it is 7.2%. In addition, the residuals of the fitted model are closer to being normally distributed for log-BMI than when fitting BMI (Supplementary Fig. 4). The s.e. of the regression coefficients from models on the log scale should therefore be better calibrated than those from models on the original scale.

If a trait follows a log-normal distribution better than a normal distribution, this may indicate that influences on the trait act multiplicatively rather than additively. This could lead to inflation of test statistics for multiplicative interaction models when fitting them on the original rather than log-transformed scale. Our analysis is therefore likely to be more conservative than those on the original, untransformed scale.

We fit models with pairwise interactions between the number of copies of the *FTO* risk allele and multiple environmental variables. We illustrate this with a simple model where *FTO* interacts with a single environmental variable, *x*, on the log scale. Formally, the model is


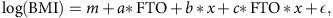


where *m* is the mean of log-BMI, *FTO* is the mean-centred number of copies of the *FTO* risk allele, *x* is the mean-centred environmental variable and *ɛ* is an independent normal error term. Transformed back onto the original BMI scale this is:


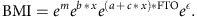


The effect of an interaction with an environmental variable *x* is to either enhance or dampen the proportional change in BMI expected with each additional copy of the *FTO* risk allele. For example, if *x* is a physical activity variable with a negative interaction with *FTO*, then for those with above average levels of physical activity, each copy of *FTO* may raise BMI by <1% on average, whereas for inactive people it may raise BMI by over 1% on average. In the analysis, we generalize this to *FTO* interacting with multiple environmental factors simultaneously, which gives the model the ability to determine whether specific environmental factors are interacting with *FTO* independently of their correlations with the other modelled environmental variables. We give an example of the model for two lifestyle factors *x* and *y* that have main effects *b* and *c*, and interaction effects *b*_FTO_ and *c*_FTO_ with FTO on BMI:


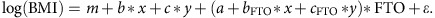


### Model selection and score construction

We constructed diet and activity scores from individuals in the United Kingdom currently without genotype data. We selected 231,906 of these individuals who had self-declared British ancestry, complete data on the relevant variables, and were known to be born in the United Kingdom or Ireland.

We fitted a joint model for log-BMI with age, sex, age^2^, age^3^, the interactions of sex with age and its square and cube, the north and east co-ordinates of birthplace, and all of the variables listed above. We include interactions with age and sex for: the activity variables, frequency of alcohol consumption, TV watching, sleep duration, current smoking, and Townsend deprivation index. We did not include interactions with age and sex for dietary variables for simplicity.

We calculated the *t*-statistic of the marginal regression coefficients in *R*, and we used cross-validation to select a *t*-statistic magnitude threshold for inclusion of the variables in the model. We used 10-fold cross-validation to select the model with the highest estimated out-of-sample *R*^2^, which was 13.01% and corresponded to a *t*-score threshold of 1.15 and a model with 42 variables. We call this model the ‘BMI' model and list its variables in Table 2.

To estimate the coefficients of the scores, we refitted the ‘BMI' model on the sample comprised of all ten folds combined, in which it had an *R*^2^ of 13.03%. We then used the coefficients from this model fit to calculate diet and activity scores for both the British and Diverse Samples. We used the calculated diet and activity scores along with the other variables kept by cross-validation and genetic information to assess evidence for interactions with FTO in the ‘Scores', ‘Activity' and ‘Diet' Models (Table 2).

### Genotype data

We used genotype data from the Interim Data Release of the UK Biobank Project. Quality control is described in the UK Biobank genotyping QC document^22^. We excluded individuals from the analysis that had been flagged as problematic by the Biobank QC.

We split the genotyped sample comprising 119,132 individuals with complete observations of the model variables into two subsamples. First, we split the sample by a variable provided by UK Biobank, indicating that an individual is genetically (very close to) British, as determined by a principal component analysis and self-reported ethnicity^22^. This produced two samples: a genetically ‘British' Sample and a genetically ‘Diverse' Sample. UK Biobank has determined genetic relatedness, and labelled pairs as related if their kinship indicated third degree or closer relatedness. UK Biobank pruned the ‘British' individuals of relative pairs, with one of each relative pair moved out of the ‘British' group.

For our analysis, it is helpful if the two subsamples are approximately independent. To achieve this, we further modified the UK Biobank groupings by moving individuals who had any genotyped third degree or closer relatives from the British Sample into the Diverse Sample. That is, while UK Biobank had removed one member of each pair of close relatives from the ‘British' Sample, we additionally removed the other individual in each pair, so that all pairs of related individuals fall in the ‘Diverse' Sample in our analysis.

We obtained genotypes for SNP rs1421085. At this SNP, 0.20% of calls are missing in the British Sample and 0.24% of calls are missing in the Diverse Sample. We excluded samples with missing calls. The frequency of the minor allele is 40.03% in the European Sample and 39.93% in the Diverse Sample.

The final British Sample has 89,552 individuals with no close relatives genotyped, and the final Diverse Sample has 29,580 individuals, containing close relatives.

Individuals in the UK Biobank interim data release were genotyped on one of two very similar genotyping chips, called the Axiom UKBiLEVE or Axiom UKBiobank array. As recommended in the UK Biobank QC documentation^22^, we include the array on which the individual was genotyped as a covariate in all analyses, and we also include the interaction of FTO with genotyping array as a variable in all the models.

### Control of population structure

Details of the population structure control procedures are contained in the Supplementary Note. Both our tests of the efficacy of population structure control (Supplementary Note) and the consistency of our estimates of the effects of *FTO* across the two samples (Fig. 1a) argue against our analysis being overly contaminated by population structure. Many other genome-wide association studies of samples taken from UK Caucasians have also shown that population structure is not a major factor^51^,^52^.

### Nutrient analysis

We took the nutrient estimates calculated by UK Biobank from the 24 h dietary recall questionnaire^41^. The variables are continuous and non-negative, with many extreme upper outliers. We therefore removed the top 1% of each variable's distribution. We used the ‘Scores' Model (Table 2) variables without the *FTO* variables or the diet score as covariates in linear models for log-BMI, the diet score, cooked vegetable intake and frequency of added salt. There were 12,747 individuals in the British Sample and 4,413 individuals in the Diverse Sample with complete observations of these variables. If effects were consistent between the British and Diverse Samples, they were combined in a fixed effects meta-analysis.

### Robustness of interaction effects

To investigate whether our results may have been confounded by associations between BMI and either diabetes or depression, we conducted a sensitivity analysis by removing 12,891 individuals who had reported seeing a psychiatrist for depression and 5,888 individuals who reported having been diagnosed as diabetic. Most estimated interaction effects were effectively unchanged. The largest change was for the interaction with activity score, where the estimate reduced from −0.19 to −0.15% per s.d. per copy of *FTO*.

If the observed interaction between *FTO* and frequency of alcohol consumption is a result of reverse causation, we would expect the interaction effect to be stronger in those who report having changed their alcohol consumption than in those that report no change in alcohol consumption. We therefore repeated the analysis separately in two subsets of the British Sample: those that answered that their alcohol intake is ‘about the same' (*n*=37,534) as it was 10 years ago and those that did not (*n*=57,193). We found no significant difference in the estimated interaction effects in the two groups (*z*=−0.02, *P*=0.49, one-sided test for stronger interaction in the group reporting change in alcohol consumption). We also found no evidence that FTO genotype affected the probability of reporting a change in alcohol consumption (*P*=0.87).

To assess whether the interaction between *FTO* and the diet score may reflect reverse causality, we repeated the analysis separately in two subsets of the British Sample: those that answered ‘no' to the question ‘Have you made any major changes to your diet in the last five years?' (*n*=33,781) and those that did not (*n*=55,675). The observed difference in effect was in the opposite direction to that predicted by reverse causation and was not significant (*z*=−0.63, *P*=0.73, one-sided test).

The effect of *FTO* on a lifestyle variable can also suggest whether reverse causation may be occurring: if *FTO* affects a lifestyle variable without control for BMI, but *FTO* does not affect it when controlling for BMI, then this indicates that *FTO* may be affecting the lifestyle variable through BMI, an instance of reverse causation. We therefore tested whether *FTO* affected variables that it may interact with: alcohol frequency, squared deviations from mean sleep duration, added salt, and the activity and diet scores (Supplementary Table 2). To do this, we regressed these variables onto *FTO*, the genotyping array and the top 20 principal components.

We found evidence that the *FTO* risk allele reduced the alcohol score (*P*=9.3 × 10^−3^) without the control for BMI, but found no evidence for this (*P*=0.68) with the control for BMI. This is consistent with the *FTO* risk allele reducing alcohol consumption as a consequence of increasing BMI, an instance of reverse causation. However, we did not find evidence that reverse causation affected the estimate of the interaction with *FTO* (above).

In contrast, we did not find any strong evidence that *FTO* affects the activity score, diet score, squared deviations from mean sleep duration or frequency of adding salt to food when not controlling for BMI. When controlling for BMI, however, there is some evidence that *FTO* affects all of these variables (Supplementary Table 2) in the direction that would be expected to decrease BMI according to the main effect of each lifestyle factor on BMI.

We used subjects' answers to the question ‘In general how would you rate your overall health?' to see whether there was evidence that the interactions between lifestyle factors and FTO were confounded with overall health. The answers were encoded as: 1, excellent; 2, good; 3, fair; and 4, poor.

It is likely that an individual's self-perception of their overall health is partially determined by their BMI. The correlation between this encoding and log-BMI was 0.27. Regressing overall health on principal components, genotyping array and *FTO* gives a statistically significant effect of *FTO* (*P*=2 × 10^−4^). However, adding log-BMI to the regression removes the evidence for the effect of *FTO* on overall health (*P*=0.29), indicating that *FTO* likely effects self-reported overall health through its effect on BMI. We therefore chose not to include self-reported overall health as a covariate in our primary analyses. Doing so would in effect have focused the primary analyses on the component of BMI remaining after regressing out any effect of BMI on self-reported overall health. This would have complicated interpretation, and in addition precluded comparison with other studies based directly on BMI.

By way of a sensitivity analysis and to assess the possibility that our results may be driven by a latent variable related to overall health, we fitted the ‘Scores' Model (Table 2) in the British Sample along with overall health and its interaction with *FTO*. We found no significant evidence that overall health interacts with *FTO* (0.14% [−0.01%, 0.30%], *P*=0.10). The other estimated interaction effects reduced slightly in magnitude: activity score (−0.19 to −0.16%), alcohol frequency (−0.28 to −0.25%), diet score (0.25 to 0.23%), squared deviations from mean sleep duration (0.13 to 0.11%) and age (−0.16 to −0.14%). We also note, from fitting the ‘Diet' Model (Table 2) with the overall health added, that the estimated interaction with frequency of added salt did not change from 0.23%.

### Data availability

All the data in this study come from the interim data release of the UK Biobank Genetic data. Applications for access can be made on the UK Biobank website: http://www.ukbiobank.ac.uk/register-apply/.

## Additional information

**How to cite this article:** Young, A. I. *et al*. Multiple novel gene-by-environment interactions modify the effect of *FTO* variants on body mass index. *Nat. Commun.* 7:12724 doi: 10.1038/ncomms12724 (2016).

## Supplementary Material

Supplementary Information Supplementary Figures 1-4, Supplementary Tables 1-2, Supplementary Note 1 and Supplementary References

## Figures and Tables

**Table 1:** Baseline characteristics of the samples.

	**British**	**Diverse**
Sample size	89,552	29,580
BMI	27.4 (4.69)	27.4 (4.82)
Age (years)	56.8 (7.93)	55.6 (8.21)
% Male	48.2%	46.1%
Copies of rs1421085 risk allele	0.801 (0.69)	0.799 (0.694)
Townsend deprivation index	−1.64 (2.9)	−0.881 (3.26)
Sleep duration (hours per night)	7.18 (1.05)	7.13 (1.11)
% Regular tobacco smoker	9%	10.1%
Hours watch TV per day	2.78 (1.57)	2.71 (1.68)
Alcohol intake frequency (0–5)	3.2 (1.47)	2.96 (1.56)
Number of days/week walk 10+ minutes	5.37 (1.96)	5.38 (1.97)
Number of days/week moderate physical activity 10+ minutes	3.59 (2.33)	3.62 (2.35)
Number of days/week vigorous physical activity 10+ minutes	1.84 (1.94)	1.91 (2)
Cooked vegetable intake (heaped teaspoons per day)	2.69 (1.57)	2.85 (1.95)
Oily fish intake*	1.64 (0.912)	1.66 (0.942)
Non-oily fish intake*	1.81 (0.765)	1.77 (0.8)
Processed meat intake*	1.92 (1.04)	1.82 (1.09)
Poultry intake*	2.32 (0.859)	2.3 (0.911)
Beef intake*	1.48 (0.821)	1.42 (0.869)
Lamb/mutton intake*	1.11 (0.694)	1.12 (0.749)
Pork intake*	1.16 (0.698)	1.11 (0.772)
Cheese intake*	2.56 (1.06)	2.51 (1.1)
Bread intake (slices per week)	12.7 (8.35)	12.3 (8.49)
Tea intake (cups per day)	3.54 (2.77)	3.4 (2.76)
Frequency of added salt (1–4)	1.64 (0.855)	1.71 (0.899)
		

The mean and s.d. (in brackets) are shown.

^*^A indicates that the variable is encoded as: 0, never; 1, less than once a week; 2, once a week; 3, 2–4 times a week; 4, 5–6 times a week; and 5, once or more daily. Alcohol intake frequency is encoded as: 0, never; 1, special occasions only; 2, one to three times a month; 3, once or twice a week; 4, three or four times a week; and 5, daily or almost daily. For the frequency of added salt, the categories are: 1, never/rarely; 2, sometimes; 3, usually; and 4, always.

**Table 2:** Summary of the variables used as predictors of BMI in each of the models.

**Model**	**BMI**	**Scores**	**Activity**	**Diet**
*Age and sex* (sex, age × sex, age^2^, age^2^ × sex, age^3^, age^3^ × sex)				
East co-ordinate		—	—	—
East co-ordinate × age		—	—	—
East co-ordinate × sex		—	—	—
FTO (rs1421085)	—			
*Activity variables* (‘Number of days/week walk 10+ minutes', ‘Number of days/week moderate physical activity 10+ minutes', ‘Number of days/week vigorous physical activity 10+ minutes', and their interactions with age and sex)		—		—
Activity variables × FTO	—	—		—
Activity score	—		—	
Activity score × FTO	—		—	
*Diet variables* (‘cooked vegetable intake', ‘non-oily fish intake', ‘oily fish intake', ‘processed meat intake', ‘poultry intake', ‘beef intake', ‘lamb/mutton intake', ‘pork intake', ‘cheese intake', ‘bread intake', ‘tea intake' and ‘frequency of added salt')		—	—	
Diet variables × FTO	—	—	—	
Diet score	—			—
Diet score × FTO	—			—
*Other variables* (‘age','alcohol intake frequency',‘sleep duration','sleep duration^2^', ‘current regular smoker (yes/no)',‘Townsend deprivation index', ‘Hours watch TV', and their interactions with age and sex)				
Other variables × FTO				
Genotyping array	—			
Genotyping array × FTO	—			

BMI, body mass index; CI, confidence interval; FTO, fat mass and obesity associated.

An ‘ × ' between two variables indicates an interaction effect. The ‘BMI' model is the model chosen by the cross-validation procedure in the non-genotyped sample (see Methods), and the ‘Scores' Model uses the coefficients fitted in the ‘BMI' model to construct the activity and diet scores. The ‘Activity' and ‘Diet' Models each have their relevant score variable replaced with the constituent variables of the score: ‘Activity score' replaced with ‘Activity variables' and so on. Note that to adjust for population structure in the models fitted in the genotyped samples, we added principal components in the British Sample, and we added random effects in a mixed model in the Diverse Sample (Methods).

**Table 3:** Summary of the variables with evidence for interactions with FTO.

**Variable**	**Estimate**	**95% CI**	***P*** **value**
	−0.19	[−0.34, −0.05]	1.0e−02
Activity score	−0.35	[−0.6, −0.1]	5.8e−03
	−0.23	[−0.36, −0.11]	3.1e−04
	−0.28	[−0.43, −0.13]	2.8e−04
Alcohol frequency	−0.12	[−0.38, 0.14]	3.6e−01
	−0.24	[−0.37, −0.11]	3.0e−04
	0.25	[0.11, 0.4]	7.0e−04
Diet score	0.43	[0.17, 0.69]	1.1e−03
	0.30	[0.17, 0.43]	5.0e−06
	0.13	[0.04, 0.22]	4.6e−03
Sleep squared	0.14	(−0.01, 0.3]	7.3e−02
	0.13	[0.06, 0.21]	8.0e−04
	0.23	[0.08, 0.38]	2.9e−03
Added salt	0.17	[−0.09, 0.44]	1.9e−01
	0.21	[0.08, 0.34]	1.2e−03

BMI, body mass index; CI, confidence interval; FTO, fat mass and obesity associated.

The table shows the estimated interaction effect with FTO expressed as the % change in BMI per copy of FTO and per s.d. of the variable. The first line for each variable gives the estimate in the British Sample, the second line gives the estimate in the Diverse Sample and the third line gives the combined estimate. ‘Sleep squared' refers to squared deviations from mean sleep duration.
