# Supplementary material for: Multiple novel gene-by-environment interactions modify the effect of FTO variants on body mass index
Source: Nat Commun. 2016 Sep 6;7:12724. doi: 10.1038/ncomms12724 (PMC5025863; doi:10.1038/ncomms12724)
Supplement: Supplementary Information — Supplementary Figures 1-4, Supplementary Tables 1-2, Supplementary Note 1 and Supplementary References [file ncomms12724-s1.pdf]

Supplementary Figures

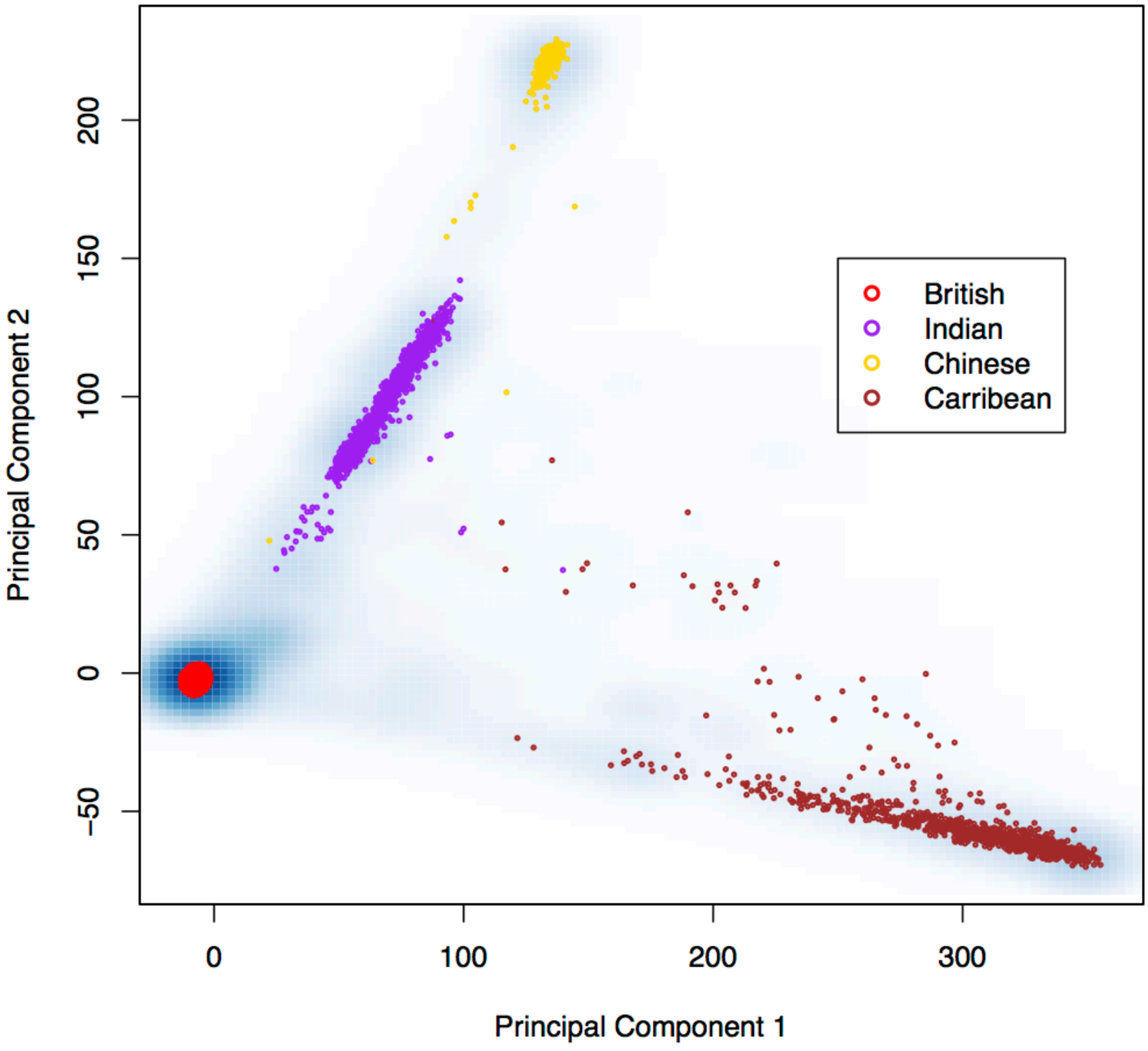

Supplementary Fig 1. Comparison of sub-samples on the first two principal components of genetic variation. The British sample is plotted with red points. The sub-samples of the diverse sample with self-declared Indian, Chinese, and Carribean ancestry are highlighted with different coloured points. The smoothed density of the diverse sample is shown in blue shading.

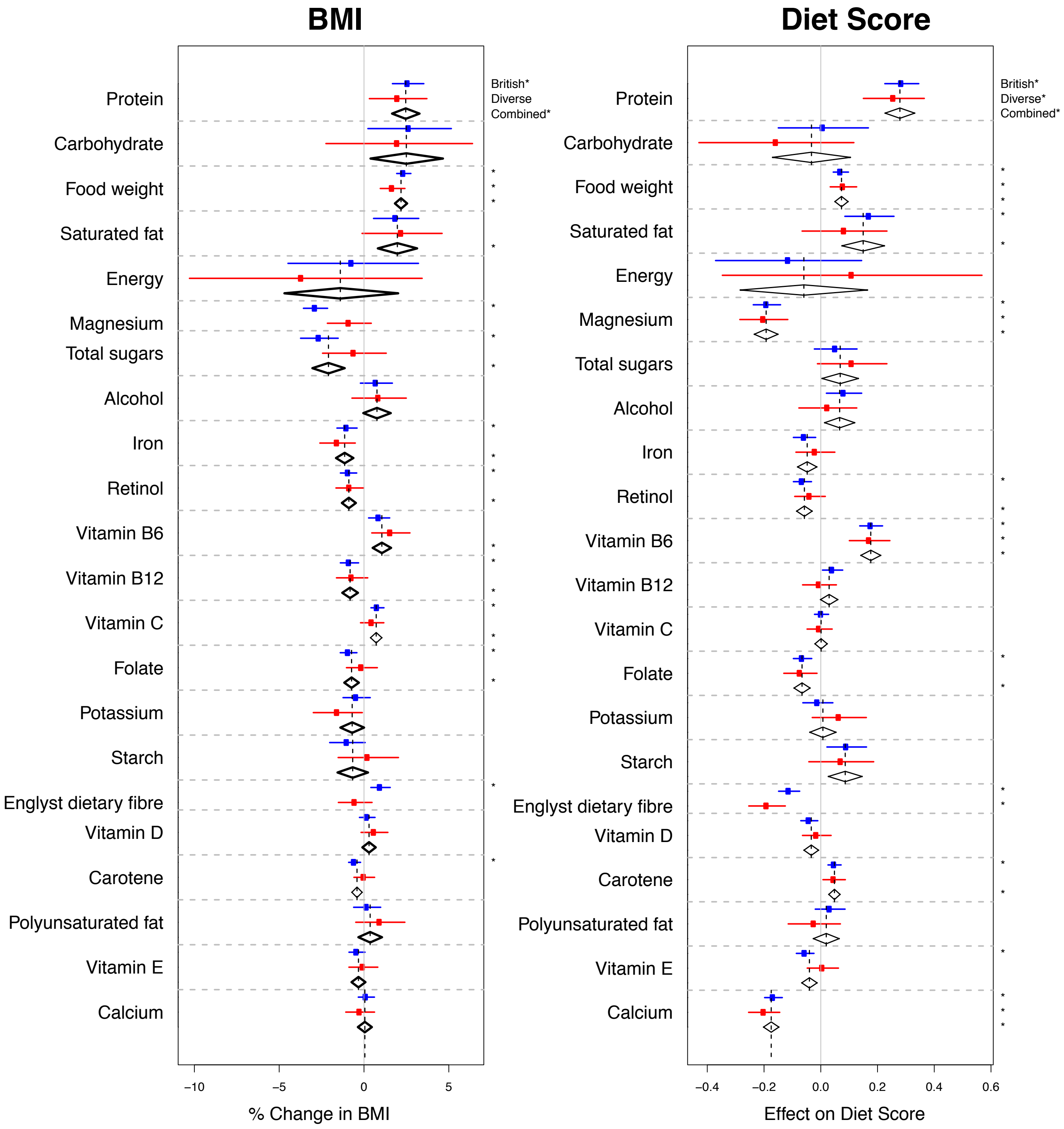

Supplementary Fig 2. The associations of different nutrient quantities with BMI and diet score. Nutrient quantities were estimated from 24 hour dietary recall. Nutrients were fitted jointly along with variables from the 'BMI' model (Table 2 and Methods). For BMI, the effects are expressed as the percentage change in BMI per standard deviation of the nutrient, and for the diet score the effect is the standard deviation change in diet score per standard deviation of the nutrient. The estimated effects and 95% confidence intervals are plotted for each sample: the British sample (n=12,747, blue) and the diverse sample (n=4,413, red). If there is no statistically significant heterogeneity ($p > 0.05$) between the samples, a combined estimate from a fixed effects meta-analysis is also plotted (diamonds). A star on the right indicates the p-value below the Bonferroni corrected significance threshold of 0.05/22.

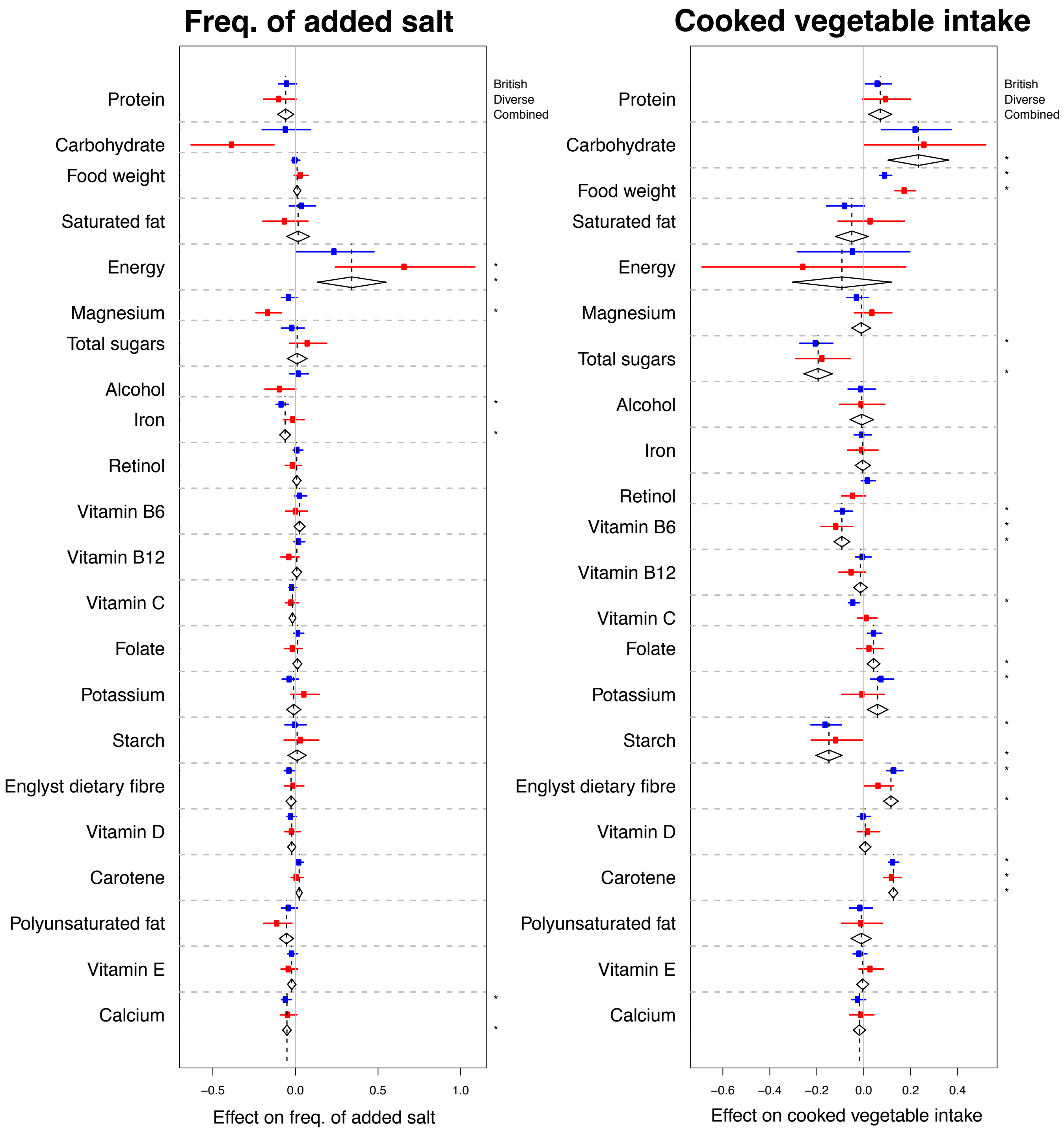

Supplementary Fig 3. The associations of different nutrient quantities with frequency of added salt and cooked vegetable intake.. Nutrients were fitted jointly along with variables from the 'BMI' model (Table 2 and Methods), excluding dietary variables and FTO. The effects are expressed as the standard deviation change per standard deviation of the nutrient. The estimated effects and 95% confidence intervals are plotted for each sample: the British sample (n=12,716, blue) and the diverse sample (n=4,418, red). If there is no statistically significant heterogeneity ($p > 0.05$) between the samples, a combined estimate from a fixed effects meta-analysis is also plotted (diamonds). A star on the right indicates the p-value below the Bonferroni corrected significance threshold of 0.05/22.

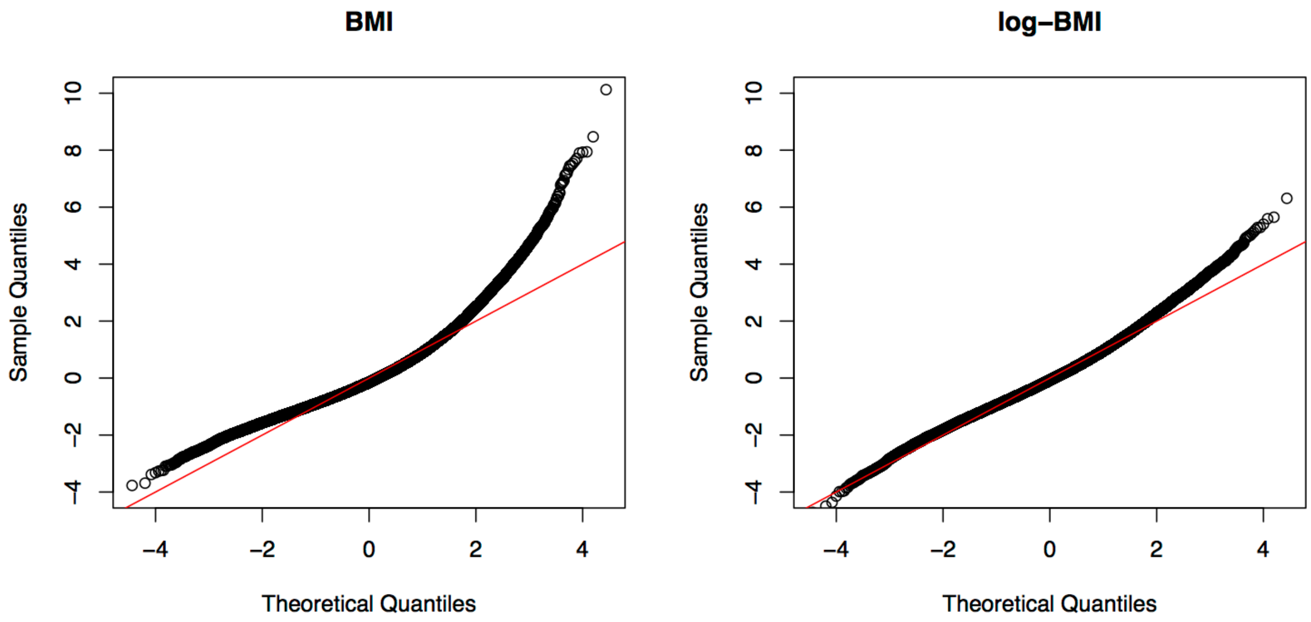

Supplementary Fig 4. Comparison of normality of BMI and log-BMI. Normal quantile-quantile plots for the residuals of the regression of BMI and log-BMI on the model variables, excluding interactions with FTO.

Supplementary Tables

Ethnicity	Percentage
British	61
Other white background	12
Irish	10
Indian	3.3
Carribean	2.6
Other	2.5
African	1.9
Any other asian background	1.0
Pakistani	1.0
Prefer not to answer	1.0

Supplementary Table 1. Composition of the diverse sample by self-declared ethnicity. Groups with 1% or greater representation are shown.

	FTO		FTO (+BMI)	
	Estimate	p-value	Estimate	p-value
Alcohol score	-0.011	4.6e-02	-0.002	0.68
Activity score	-0.001	7.6e-01	0.009	0.05
Diet score	0.003	6.2e-01	-0.012	0.03
Frequency of added salt	-0.007	2.3e-01	-0.012	0.03
Sleep Squared	-0.008	4.8e-01	-0.017	0.03

Supplementary Table 2. The effect of FTO on selected variables. Column 1 gives the estimated effect of FTO on the variable (expressed as SD change in response per copy of FTO), and column 2 gives the assocaited p-value. Columns 3 and 4 give the same when also fitting log-BMI as a covariate. See Methods for analysis details.

Supplementary Note 1

Control of Population Structure

For the British sample, we calculated principal components from the sample determined to be genetically British by UK Biobank. We LD-pruned SNPs using PLINK in a sliding window of size 1000 to ensure that no pair of SNPs within the window had an R^2 of more than 0.1. We filtered out SNPs with minor allele frequency less than 0.05, missingness greater than 1%, and Hardy-Weinberg exact test p-value less than 10^{-6}. This left ~104,000 SNPs across the genome. We used EIGENSOFT[1] with 'fastmode'[2] on to calculate the top 20 principal components. We fitted the 'Scores', 'Activity', 'Alcohol', and 'Diet' models (Table 2) using *R*[3], with the top 20 principal components added.

For the diverse sample, we used a mixed model to prevent confounding due to family relatedness and population structure not captured by principal components[4–7]. We filtered out SNPs with minor allele frequency less than 0.01, with more than 1% missing calls, and Hardy-Weinberg equilibrium exact test p-value less than 10^{-10}. We used a stronger threshold for the Hardy-Weinberg equilibrium exact test for the diverse sample because, while we wanted problematic SNPs with gross violations of equilibrium to be removed, Hardy Weinberg equilibrium is not expected to hold exactly in ethnically mixed samples. To fit the models in the diverse sample, we used a mixed model with two random effects: one from the SNPs on chromosomes other than 16, and one from the SNPs on chromosome 16 more than 2cM away from rs1421085, where genetic distance was determined using the genetic map provided by UK Biobank. We calculated genetic relatedness matrices using GCTA[8], and fitted the models using the Average Information algorithm in GCTA. These correspond to the maximum likelihood estimates of the fixed effects given the variance components that maximize the restricted likelihood.

Efficacy of Population Structure control

If population structure has been controlled effectively and there are no true causal loci, then the association test statistics at independent SNPs across the genome should be sampled from the null distribution. A common measure of effectiveness of control of population structure is the inflation factor[9]: this estimates the ratio of the median test statistic across the genotyped variants to the median that would be expected from the null distribution of

test statistics [9]. A weakness of this measure is that if a trait has many causal variants, which BMI is known to have[10,11], then the inflation factor should be greater than 1 even if population structure has been controlled for perfectly[12]. In the following, we calculate inflation factors for SNPs across the genome to measure how effective our control of population structure is in both samples.

To test whether a mixed model could control for the kind of structure in the diverse sample, we used BOLT-LMM[13], with the LMM-Inf setting, to calculate association statistics between log-BMI and the SNPs on the chromosomes other than 16, which contains the *FTO* locus. We used the 'BMI' model (Table 2) variables as fixed effects, excluding any interactions with *FTO*. We used BOLT-LMM instead of GCTA because of the greater computational efficiency. (Note that for this analysis we undertake association analyses at SNPs genome-wide, whereas our primary analyses are focused on a single *FTO* SNP.) The results should be comparable because BOLT-LMM with the LMM-inf setting fits the same infinitesimal mixed model as GCTA. The inflation factor over the tested chromosomes was 1.07, which is lower than 1.09 reported for a BMI meta-analysis[12].

We measured how effective adjusting for the top 20 principal components in the British sample was at controlling population structure by computing association statistics for a sample of SNPs across the genome. To ensure the association test statistics were comparable to our *FTO* analysis, we used the same code and model within *R* as for the primary analysis. However, this imposed computational constraints, preventing a genome wide analysis. We therefore selected 100 SNPs from each chromosome, leaving a gap of 100 genotyped SNPs between each selected SNP. We kept those with minor allele frequency >5% and missingness <1%, leaving 872 SNPs. We used the 'Scores' model (Table 2) with all of the *FTO* variables removed and replaced with the test SNP. The inflation factor was 1.12. While the inflation factor is higher than in the diverse sample, it is close to the inflation factor of 1.09 reported for a BMI meta-analysis[12].

Supplementary References

1. Patterson, N., Price, A. L. & Reich, D. Population structure and eigenanalysis. *PLoS Genet.* **2,** 2074–2093 (2006).

2. Galinsky, K. J. *et al.* Fast principal components analysis reveals independent evolution of ADH1B gene in Europe and East Asia. **23,** (2015).

3. R Core Team. R: A Language and Environment for Statistical Computing. (2015). at <http://www.r-project.org/>

4. Astle, W. & Balding, D. J. Population Structure and Cryptic Relatedness in Genetic Association Studies. *Stat. Sci.* **24,** 451–471 (2009).

5. Zhou, X. *et al.* Mixed linear model approach adapted for genome-wide association studies. *Nat. Genet.* **42,** 355–60 (2010).

6. Zhang, Z. *et al.* Mixed linear model approach adapted for genome-wide association studies. *Nat. Genet.* **42,** 355–60 (2010).

7. Lippert, C. *et al.* FaST linear mixed models for genome-wide association studies. *Nat. Methods* **8,** 833–5 (2011).

8. Yang, J., Lee, S. H., Goddard, M. E. & Visscher, P. M. GCTA: a tool for genome-wide complex trait analysis. *Am. J. Hum. Genet.* **88,** 76–82 (2011).

9. Devlin, B. & Roeder, K. Genomic control for association studies. *Biometrics* **55,** 997–1004 (1999).

10. Speliotes, E. K. *et al.* Association analyses of 249,796 individuals reveal 18 new loci associated with body mass index. *Nat. Genet.* **42,** 937–948 (2010).

11. Yang, J. *et al.* Ubiquitous polygenicity of human complex traits: genome-wide analysis of 49 traits in Koreans. *PLoS Genet.* **9,** e1003355 (2013).

12. Bulik-Sullivan, B. K. *et al.* LD Score regression distinguishes confounding from polygenicity in genome-wide association studies. *Nat. Genet.* **47,** 291–295 (2015).

13. Loh, P., Tucker, G., Bulik-sullivan, B. K. & Vilhj, B. J. Efficient Bayesian mixed model analysis increases association power in large cohorts Efficient Bayesian mixed model analysis increases association power in large cohorts. *Nat. Publ. Gr.* **47,** 0–79 (2014).
